# Soft Fruit Traceability in Food Matrices using Real-Time PCR

**DOI:** 10.3390/nu1020316

**Published:** 2009-12-23

**Authors:** Luisa Palmieri, Elisa Bozza, Lara Giongo

**Affiliations:** Fondazione Edmund Mach, IASMA Research and Innovation Centre, Genomics and Crop Biology Area, Via E. Mach 1, 38010, San Michele all’Adige (TN), Italy; Email: elisa.bozza@iasma.it (E.B.); lara.giongo@iasma.it (L.G.)

**Keywords:** soft fruit, traceability, real-time PCR, strawberry, blueberry, currant, raspberry, orange, pineapple

## Abstract

Food product authentication provides a means of monitoring and identifying products for consumer protection and regulatory compliance. There is a scarcity of analytical methods for confirming the identity of fruit pulp in products containing Soft Fruit. In the present work we have developed a very sensible qualitative and quantitative method to determine the presence of berry DNAs in different food matrices. To our knowledge, this is the first study that shows the applicability, to Soft Fruit traceability, of melting curve analysis and multiplexed fluorescent probes, in a Real-Time PCR platform. This methodology aims to protect the consumer from label misrepresentation.

## 1. Introduction

It is well known that soft fruit species contain numerous bioactive compounds that are beneficial to human health and well-being and may reduce the risk of disease by beneficially targeting body functions [1]. For this reason, these fruits can be used in varying proportions as ingredients in so-called Functional Foods [2]. 

Consumers are frequently attracted by the beneficial effects described on the label of such foods, therefore labels can be an important educational tool in helping consumers to make healthy food choices. Food labeling and traceability are regulated by EU directive 2000/13/EC, and subsequent amendments [3], and EU regulation 178/2002 [4], respectively. However, in some cases there is either accidental or fraudulent substitution [5]. Substitution represents not only an attempt to deceive the consumer but may also go hand in hand with dangerous practices [6]. Furthermore, there are increasing concerns regarding the presence of allergens in food products [7]. The ability to detect misrepresentation and deliberate adulteration is therefore essential for protecting the consumer.

Several modern analytical techniques are able to determine the plant or animal species present in a given food product [8,9,10]. In particular, techniques based on DNA analysis have become routine in the need to identify raw food materials [11] and could complement the chemical pool of methods that are frequently ineffective in processed food products due to the complex nature of the food matrices. Moreover, DNA is more resistant to food processing then chemical and biochemical compounds [12].

The pulps of soft fruits are widespread and essential ingredients in juices, jams, baby foods, snacks and yogurts. In the majority of these products the percentage of fruit they contain is declared. The widening market of these products has led to speculation that they may contain artificial aromas and be adulterated and mislabeled. At the moment, there is a scarcity of methods for confirming the identity of berry pulps in products containing fruit. These methods are based on HPLC [13], SPME-CG [14] and UPLC-MS/MS [15] chemical analyses or they are molecular-genetic procedures based on PCR and sequencing techniques, such as CAPS (Cleavable Amplifiable Polymorphic Sites) and Pyrosequencing® analyses [16]. However, these types of analysis are problematic for various reasons. Firstly, there are problems with the chemical methodologies employed in the product’s manufacturing system and storage. The CAPS approach is also problematic as it is based on mitochondrial DNA analysis, which in plants is heterogeneous and poorly characterized, while Pyrosequencing® is based on the analysis of SNPs on *rbc*L (ribulose biphosphate carboxylase large subunit) sequences and these being chloroplast genes the copy number varies in different tissues and different species [17]. The selection of appropriate target sequences is, therefore, important for successful quantitative analysis. The 5S DNA sequence is a suitable alternative plant sequence as it is present in a high copy number. The 5S rRNA gene sequence is highly conserved between plant species while the spacer is species-specific; the sequence has been used for phylogenetic studies [18], species identification [19] and also in a traceability study [20].

In real-time PCR, using primer pairs developed from the 5S rRNA sequence [21] and on ANS (anthocyanidin synthase) sequences [22] we can discriminate between five different berry genera and species and between these fruits and other fruit species mixed together in different types of fruit-based food products. In addition, we obtained new sequences for some of the fruit species analyzed and these were used to design new primers and fluorescent probes which were useful for quantitative analysis.

## 2. Results and Discussion

The DNA of raspberry, blueberry, blackberry, redcurrant and strawberry as well as other fruits was extracted using a commercial kit from nine “home made” juices, from 14 juice mixes (30/70%, 50/50%, 70/30%) and from 14 commercial food products. The amounts of total DNA obtained ranged between 0.003 and 1.4 μg for juices, and between 0.005 and 0.182 μg for food products (Table 1).

**Table 1:** Sample list and quantity of DNA extracted.

Home Made Juice	Composition	DNA extracted/400 µL ^*^
Pineapple	100% pineapple	0.063 μg
Orange	100% orange	0.346 μg
Apple	100% apple	1.400 μg
Raspberry	100% raspberry	0.543 μg
Redcurrant	100% redcurrant	0.008 μg
Blueberry	100% blueberry	0.006 μg
Strawberry	100% strawberry	0.043 μg
Red Orange	100% red orange	0.157 μg
Blackberry	100% blackberry	0.246 μg
**Juice Mixes**	**Composition**	DNA extracted/400 µL *
Blueberry-Orange	70%–30%	0.150 μg
Blueberry-Orange	50%–50%	0.178 μg
Blueberry-Orange	30%–70%	0.164 μg
Pineapple-Strawberry	70%–30%	0.026 μg
Pineapple-Strawberry	50%–50%	0.026 μg
Pineapple-Strawberry	30%–70%	0.145 μg
Redcurrant-Blueberry	70%–30%	0.007 μg
Redcurrant-Blueberry	30%–70%	0.003 μg
Redcurrant-Pineapple	70%–30%	0.027 μg
Redcurrant-Pineapple	30%–70%	0.011 μg
Strawberry-Blueberry	70%–30%	0.183 μg
Strawberry-Blueberry	30%–70%	0.032 μg
Orange-Strawberry	70%–30%	0.087 μg
Orange-Strawberry	30%–70%	0.356 μg
**Commercial products**	**Declared Composition**	**DNA extracted/350 mgº**
Blackberry Yogurt	not declared	0.025 μg
Apple Yogurt	of 15% fruit content: 65% apple	0.019 μg
Strawberry Jam	100% strawberry	0.085 μg
Apple/blueberry baby food	74% apple, 15% blueberry	0.067 μg
Raspberry Jam	100% raspberry	0.125 μg
Blueberry Jam	100% blueberry	0.182 μg
Snack with Strawberry Jam	not declared	0.107 μg
Blackcurrant Juice	minimum 25% blackcurrant	0.005 μg
Soft Fruit Juice	30% fruit content: 16% apple, 5% strawberry, 5% blackcurrant, 4% blackberry	0.021 μg
Blueberry/Grape Juice	55% blueberry, 45% wine grapes	0.002 μg
Blackcurrant Jam	40% blackcurrant	0.151 μg
Soft Fruit Yogurt	not declared	0.046 μg
Red Fruit Juice	18% orange, 14% apple, 3% cranberry	0.021 μg
Mixed Juice	24% blueberry	not determined

^*^DNA was extracted starting from 400 µL of juice

^°^DNA was extracted starting from 350 mg of commercial product

The extraction methods may have unpredictable effects on discriminative PCRs and there is a risk of misidentification, therefore DNA integrity assessment is necessary in order to ensure that the subsequent analysis with molecular markers is accurate [23]. To achieve this, 10 new degenerate primers were designed on conserved regions of the rbcL sequence after alignment of rbcL sequences from apple (X69749.1), blackcurrant (L11204.2), blueberry (L12625.2, AF419837.1, AF419836.1, AF419835.1, AF421107.1, AF124576.1), raspberry (U06825.1), strawberry (U06805.1), orange (58678-60105), pineapple (L19977.1) and pomegranate (L10223.1) (Table 2). Nine of these primers were used in different combinations to amplify DNA extracted from the juices and the resulting amplification products had a range of 1,000 to 100 pb. Six of these nine were used to amplify DNA derived from commercial food products. In the latter case the amplification limit was between 250 and 1,000 bp.

**Table 2:** Sequences and matching of rbcL primers.

Primer name	Sequence	Match with
rbcL1 forward	5’-TTGGCAGCATTYCGAGTAACTCC-3’	
rbcL2 forward	5’-TGGCAGCATTYCGAGTAACTC-3’	
rbcLA reverse	5’-CCTTTRTAACGATCAAGRC-3’	rbcL1 forward
rbcLB reverse	5’-AACCYTCTTCAAAAAGGTC-3’	rbcL1 forward
rbcLC reverse	5’-TTCSGCACAAAATAMGAAACGG-3’	rbcL1 forward
rbcLD reverse	5’-TAGTATTTGCDGTGAATCCC-3’	rbcL1 forward/rbcL2 forward
rbcLE reverse	5’-TGATCTCCACCAGACAKACG-3’	rbcL1 forward/rbcL2 forward
rbcLF reverse	5’-ATATGCCAAACRTGRATACC-3’	rbcL1 forward/rbcL2 forward
rbcLH reverse	5’-ATATGCCAAACRTGRATACC-3’	rbcL1 forward

**Figure 1:**
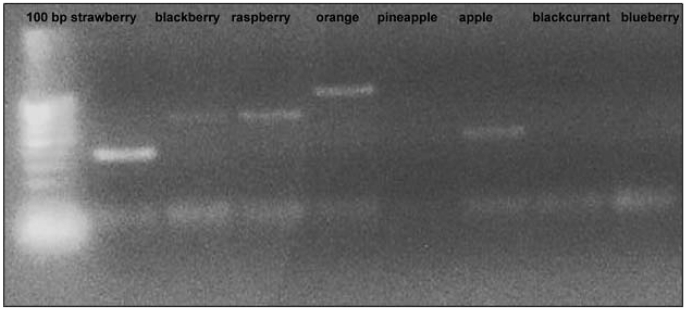
Amplification profile of some DNA samples extracted from “home-made” fruit juices observed with EMFxaANS primers.

**Figure 2:**
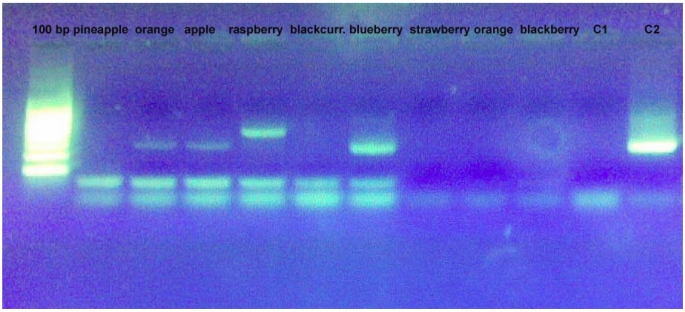
Amplification profile of some DNA samples extracted from “home–made” fruit juices observed with PI and PII primers.

**Figure 3:**
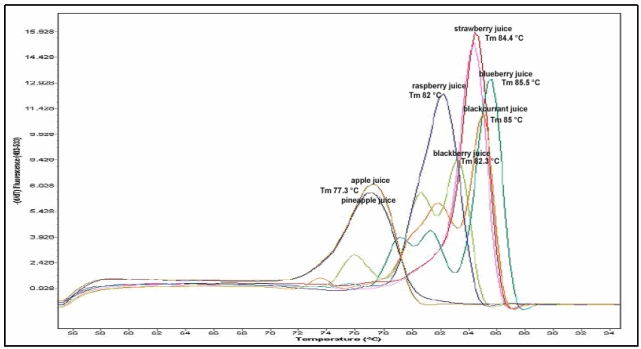
Melting peaks resulting from amplification with EMFxaANS primers showing the different melting temperatures for different fruit juices: pink line - strawberry juice, Tm 84.4 °C; light blue line - blueberry juice, Tm 85.5 °C; blue line - raspberry juice, Tm 82 °C; orange line - blackcurrant juice, Tm 85 °C; green line - blackberry juice, Tm 82.3 °C; brown and dark blue lines - pineapple juice and apple juice, Tm 77.3 °C.

To discriminate between different fruit species, it is necessary to detect sequence-specific amplification products, as in the detection of GMOs [24] or other raw materials (hazelnut, olive oil, solanaceae) [25,26,27]. Both primer pairs used in these analyses detected polymorphic profiles that were able to discriminate between our species. Using primer pairs designed from ANS fragments it was possible to discriminate between strawberry, raspberry/blackberry, apple and orange. Using primers PI and PII designed on the 5S rRNA sequence, polymorphic bands were detected in pineapple, orange, apple, raspberry, blueberry and strawberry. Both qualitative analyses were done using the simple PCR technique (Figure 1 and Figure 2) on a real-time PCR platform (Figure 3). In the second case, the fruit species were discriminated using the specific melting temperature of amplicon picks obtained with EvaGreen® fluorescent DNA staining.

Using EMFxaANS and PI/PII primers for some fruit species revealed the presence of more than one band on the agarose gel, which was supported by the presence of two peaks in real-time PCR. In these cases, it was necessary to sequence single bands cut from the gel. The identity of the polymorphic cut gel bands was determined after purification and sequencing. To determine homologies, with other ANS or 5S sequences of other plant species, the sequencing analysis products were evaluated by a BLAST search within the NCBI GenBank. New sequences were obtained for blueberry, pineapple, raspberry and orange. The new primers designed from these sequences (Table 3) gave us small specific amplification products that were useful for qualitative analyses of “home-made” juices and commercial products. 

**Table 3:** Sequences and melting temperatures of primers designed on new sequences.

Name	Sequence	Melting temp. (°C )
Orange forward	5’-GGCACGGGTTAAGTAGATTTGC-3’	60.3
Orange reverse	5’-TTATATGTTCGCGCTGGTATGATC-3’	57.1
Blueberry forward	5’-CGACCTTGGCGGAAAACA-3’	56.0
Blueberry reverse	5’-AAGTGAGTTCCCTCCACTTTCG-3’	60.0
Pineapple forward	5’-GGAGGAGCCCGAAAAACG-3’	58.2
Pineapple reverse	5’-TTTCCGCCTTCTCAAGCAGTT-3’	57.9
Strawberry forward	5’-CGAAAGGGCAAGGAAAAATG-3’	55.3
Strawberry reverse	5’-GCTCCTCCCGAGCTCATCT-3’	61.0

**Figure 4:**
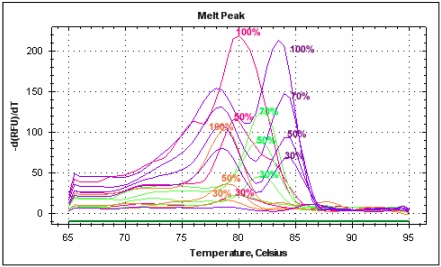
Melting peak profiles of real-time PCRs carried out on mixed juices with the new species-specific primer pairs.

**Figure 5:**
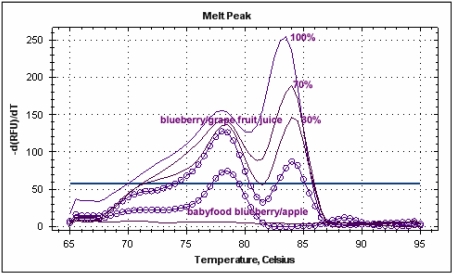
Melting peak profiles of real-time PCR carried out with blueberry juice at different percentages and blueberry-based foods with the new species-specific primer pairs.

With these new primers final qualitative and “relative quantitative” analyses were carried out on DNA extracted from pure, single-species juices, juices mixed in different proportions and commercial products with declared percentages (Figure 4 and Figure 5). We carried out a regression analysis on our “relative standards” based on fluorescences detected at different percentages and obtained R^2^ values ranging from 0.99 for pineapple to 0.93 for strawberry. 

Blueberry “relative standard” was used to compare DNA extracted from blueberry/apple baby food and blueberry/wine grape juice. The juice mix was found to have a blueberry content of 34% and the baby food 25%. The result obtained with commercial food confirmed the transferability and robustness of the protocol developed with fresh juices.

**Figure 6:**
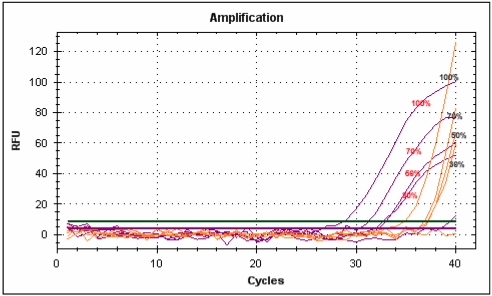
Multiplex real-time PCR carried out with dual labeled probes on DNA extracted from blueberry and orange juices mixed at different percentages.

A preliminary result was obtained using specific primers and two probes designed on blueberry and orange sequences. These two probes were found to have good specificity, even when used on mixed and multiplexed juices (Figure 6). The Multiplex PCR procedure is a useful method for the simultaneous detection of different species in one reaction and has been successfully applied to GMO detection in food [28]. In the future, these probes will be used to optimize quantitative analyses following the development of a quantitative internal standard using a Duplo-Target Plasmid Calibrator [29].

This research achieved its major objective, which was to develop a preliminary protocol to trace the presence of different berries and other fruits in simple and more complex food matrices. It was clearly demonstrated that a sufficient quantity of fruit DNA can be obtained from home-made and commercial food products. These DNAs can be successfully used for analyses on highly sensitive high-throughput platforms such as Real-Time. As is well-known, quantitative determinations carried out with EvaGreen® or SyberGreen® chemistry are influenced by variations in the efficiency of amplifications determined by primer sequences and the detection of fluorescence at the amplification plateau and may be good indicators for subsequent absolute quantitative analysis. The development of specific fluorescent probes is a good point of departure in the short term, given that these represent an absolute and highly sensitive quantitative protocol able to detect the presence of the fruits in question even in very low quantities, as has been demonstrated in other published works on determining the presence of GMOs in human and animal foods [30,31]. Finally, the new sequences obtained for some of these small fruits can be used to improve knowledge of these plant species through the enhancement of new molecular markers based on detection of single nucleotide polymorphisms (SNPs) able to discriminate between the species and varieties. 

## 3. Experimental Section

### 3.1. Food Samples and DNA Extraction

DNA was extracted starting from 400 µL of nine “home-made” fruit juices, from 400 µL of 14 mixes of these juices and from 350 mg of 14 food products containing soft fruits (Table 1) using GreesDNAFoodKIT^®^ (InCura). DNA quantification was carried out using a Qbit-fluorimeter^®^ (Invitrogen). 

### 3.2. DNA Integrity Evaluation

DNA integrity was assessed using capillary electrophoresis on Experion^®^ DNA-Chip (Biorad) and by PCR analysis with *rbc*L primer pairs developed on a ribulose biphosphate carboxylase large subunit (*rbc*L) sequence that produced amplicons with increasing length. PCRs were performed in 25 µL volume containing: ≅ 30 ng of DNA, 1X reaction buffer, 0.1 mM dNTPs, 0.6 µM of each possible pair of primers (Table 2) and 1.75 U of Hot Start Taq Polymerase DNA with the following profile: 95 °C for 7 min, 35 cycles at 95 °C for 15 sec, 56 °C for 30 sec, 72 °C for 30 sec; final extension at 72 °C for 10 min. The PCR products were visualized on 1.5% GelStar^TM^ Nucleic Acid Stain (Lonza, Milan, Italy) stained agarose gel on transilluminator and on Experion^®^ DNA-chip.

### 3.3. PCR Analysis

Basic PCR analyses were carried out using primer pairs designed on ANS and 5S rRNA sequence regions. PCRs were performed from EMFxaANS primers in a final volume of 25 µL containing: ≅ 30 ng of DNA, 1x PCR Buffer, 0.2 mM dNTPs, 0.5 µM of each primer and 1.25 U of HotStart Taq Polymerase using the thermal profile reported in the published work [22]; for degenerated primers designed on 5S-NTS region in a final volume of 20 µL containing: ≅ 30 ng of DNA, 1X PCR Buffer, 0.2 mM dNTPs, 0.2 µM of each primer and 0.8 U of HotStart Taq Polymerase with the following thermal profile: 95°C for 5 min, Touch Down PCR 10 cycles at 95 °C for 45 sec, from 68 to 63 °C (decreasing 0.5°C with each cycle) for 45 sec, 72 °C for 1 min, 35 cycles at 95 °C for 45 sec, 63 °C for 45 sec, 72°C for 1 min, final extension at 72 °C for 7 min. DNA was extracted from the leaves of the various berry plants analyzed as an amplification control.

### 3.4. Sequencing Analysis

Polymorphic PCR fragments derived from amplification with ANS and PI, PII primers were cut and purified using the commercial kit GreesDNA-KitCleanOut® (InCura). Purification products were sequenced using a BigDie 3.1 ABI kit according to manufacturer’s specifications.

### 3.5. Qualitative and Quantitative Real-Time PCR

The primers used in the basic PCR analyses were used to evaluate the different melting temperatures of the amplicons obtained by Real Time PCRs performed on Roche LightCycler® (analysis with EMFxaANS primers) and Bio-Rad CFX® (analysis with 5S-primers) Real-Time platforms using, respectively, SYBRGreen® (ABI) and EvaGreen® (Biorad) according to manufacturers’ protocols. New primers and probes were designed on new sequences of blueberry, orange, pineapple, strawberry and raspberry using Primer Express software (ABI) (Table 3). A real-time PCR using the new primer pairs and an EvaGreen® kit was carried out on DNA extracted from: 100% orange juice, 100% blueberry juice, 100% strawberry juice, 100% pineapple juice, mixes of these juices in varying proportions (30/70%, 50/50%, 70/30%) and commercial products with declared percentages (blueberry jam, one baby food, blueberry/wine grape juice, mixed juice, strawberry jam, soft fruit juice) with the following thermal profile: 95 °C for 3 min, 40 cycles at 95 °C for 10 sec, 52 °C for 10 sec, 72 °C for 30 sec, 1 cycle at 95 °C for 10 sec, 55 °C for 30 sec, a melt curve from 65 °C to 95 °C with an increment of 0.5 °C every 0.05 sec.

A preliminary analysis in simple and multiplex PCR was carried out with new primer pairs specific to blueberry and orange and with the fluorescent probes 5’-Cy5-AACCACGTGCCTTGG-EclipseQuencer-3’ and 5’-HEX-TGCACATGCTGATGGG-EclipseQuencer-3’ (Eurofins MGW Operon), respectively, using IQ Mastermix (Biorad) according to manufacturer’s specifications and with the following thermal profile: 95 °C for 3 min, 40 cycles at 95 °C for 10 sec, 51 °C for 10 sec, 72 °C for 30 sec. The plate was read at the end of each cycle.

## 4. Conclusions

Under EU law [4], ““*traceability” means the ability to trace and follow a food, feed, food-producing animal or substance intended to be incorporated into a food or feed, through all stages of production, processing and distribution*” and “*Food law shall aim at the protection of interests of consumers and shall provide a basis for consumers to make informed choices in relation to the foods they consume”*. To safeguard the food industry, growers, distributors and consumers from fraud and satisfy the increasing high-throughput demands of the food industry, new analytical methodologies able to make more specialized and accurate measurements are needed. These methodologies must focus on performance, sensitivity, reliability, simplified use and routine assays. 

The preliminary analytical method that we have developed and are improving aims to detect and quantify the presence of soft fruits along the food chain “from farm to fork” and, as a consequence, aims to increase the demand for raw materials from the growers by the food industry and help the consumer in product choice. Improving knowledge of berry sequences and species-specific probe numbers will increase the power of this traceability method.
